# Tris(ethane-1,2-diamine-κ^2^ *N*,*N*′)cobalt(III) carbonate iodide tetra­hydrate

**DOI:** 10.1107/S1600536810033143

**Published:** 2010-08-21

**Authors:** Greg Brewer, Ray J. Butcher, Jerry P. Jasinski

**Affiliations:** a Department of Chemistry, Georgetown University, 620 Michigan Av. NE, Washington, DC 20064, USA; b Department of Chemistry, Howard University, 525 College St. NW, Washington, DC 20059, USA; c Department of Chemistry, Keene State College, 229 Main Street, Keene, NH 03435-2001, USA

## Abstract

The title compound, [Co(C_2_H_8_N_2_)_3_](CO_3_)I·4H_2_O, crystallizes with a [Co(en)_3_]^3+^ cation (en is ethane-1,2-diamine), CO_3_^2−^ and I^−^ anions and four water mol­ecules in the asymmetric unit. In the cation, the three rings formed by the ethyl­enediamine units and the Co^III^ metal ion are in slightly distorted twist conformations. Numerous O—H⋯O, N—H⋯O, N—H⋯I and O—H⋯I inter­molecular hydrogen bonds between the cation and two anions in concert with the four water mol­ecules dominate the crystal packing and create a supra­molecular infinite three-dimensional framework.

## Related literature

For background to double salts, see: Dvorkin *et al.* (1989 ▶, 1991 ▶); Farago *et al.* (1967 ▶). Brewer & Butcher (2009 ▶). For the synthesis, see: Broomhead *et al.* (1960 ▶). For hydrolysis of cyanate to give carbonate at elevated temperatures, see: Seifer & Tarasova (1982 ▶); Seifer *et al.* (1981 ▶); Piazzesi *et al.* (2007 ▶). For thermodynamics of the outer sphere solution inter­action of [Co(en)_3_]^3+^ with the carbonate ion, see: Mironov *et al.* (1973 ▶, 1976 ▶). For related structures containing the [Co(en)_3_]^3+^ cation, see: Brouty *et al.* (1976 ▶); Liu *et al.* (1995 ▶); Lappin *et al.* (1993 ▶); Mizuta *et al.* (1988 ▶).
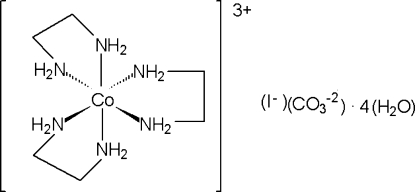


## Experimental

### 

#### Crystal data

[Co(C_2_H_8_N_2_)_3_](CO_3_)I·4H_2_O
*M* *_r_* = 498.22
Orthorhombic, 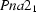

*a* = 16.6907 (2) Å
*b* = 8.7031 (1) Å
*c* = 12.5718 (2) Å
*V* = 1826.19 (4) Å^3^
*Z* = 4
Mo *K*α radiation
μ = 2.67 mm^−1^
*T* = 123 K
0.52 × 0.46 × 0.35 mm

#### Data collection

Oxford Diffraction Gemini R diffractometer
Absorption correction: multi-scan (*CrysAlis RED*; Oxford Diffraction, 2007 ▶) *T*_min_ = 0.737, *T*_max_ = 1.000
25329 measured reflections
7440 independent reflections
6109 reflections with *I* > 2σ(*I*)
*R*_int_ = 0.028

#### Refinement

*R*[*F* ^2^ > 2σ(*F* ^2^)] = 0.023
*wR*(*F* ^2^) = 0.048
*S* = 0.97
7440 reflections
224 parameters
13 restraints
H atoms treated by a mixture of independent and constrained refinement
Δρ_max_ = 0.50 e Å^−3^
Δρ_min_ = −0.52 e Å^−3^
Absolute structure: Flack (1983 ▶), 3466 Friedel pairs
Flack parameter: 0.034 (8)

### 

Data collection: *CrysAlis PRO* (Oxford Diffraction, 2007 ▶); cell refinement: *CrysAlis PRO*; data reduction: *CrysAlis PRO*; program(s) used to solve structure: *SHELXS97* (Sheldrick, 2008 ▶); program(s) used to refine structure: *SHELXL97* (Sheldrick, 2008 ▶); molecular graphics: *SHELXTL* (Sheldrick, 2008 ▶); software used to prepare material for publication: *SHELXTL*.

## Supplementary Material

Crystal structure: contains datablocks global, I. DOI: 10.1107/S1600536810033143/bt5324sup1.cif

Structure factors: contains datablocks I. DOI: 10.1107/S1600536810033143/bt5324Isup2.hkl

Additional supplementary materials:  crystallographic information; 3D view; checkCIF report

## Figures and Tables

**Table 1:** Hydrogen-bond geometry (Å, °)

*D*—H⋯*A*	*D*—H	H⋯*A*	*D*⋯*A*	*D*—H⋯*A*
N11—H11*A*⋯O3*S*^i^	0.92	1.90	2.7625 (19)	155
N11—H11*B*⋯I^ii^	0.92	3.08	3.8409 (14)	141
N12—H12*A*⋯O2*S*^iii^	0.92	1.93	2.8042 (19)	158
N12—H12*B*⋯O1*W*	0.92	2.27	3.020 (2)	138
N21—H21*A*⋯O1*S*^i^	0.92	2.10	2.9891 (18)	161
N21—H21*B*⋯I	0.92	2.80	3.6165 (13)	149
N22—H22*A*⋯O2*W*	0.92	2.06	2.970 (2)	172
N22—H22*B*⋯O3*S*	0.92	1.91	2.8104 (19)	166
N31—H31*A*⋯O3*W*	0.92	2.01	2.907 (2)	165
N31—H31*B*⋯O2*S*	0.92	1.92	2.821 (2)	165
N32—H32*A*⋯O1*S*^iii^	0.92	2.12	2.9760 (18)	155
N32—H32*A*⋯O2*S*^iii^	0.92	2.61	3.146 (2)	117
N32—H32*B*⋯I^ii^	0.92	2.80	3.6456 (13)	153
O1*W*—H1*W*1⋯O4*W*^iv^	0.81 (2)	2.19 (3)	2.892 (3)	146 (4)
O1*W*—H1*W*2⋯O3*W*^v^	0.79 (2)	2.07 (2)	2.805 (3)	156 (3)
O2*W*—H2*W*1⋯O1*S*^iii^	0.79 (2)	1.90 (2)	2.6775 (17)	170 (2)
O2*W*—H2*W*2⋯O1*W*	0.81 (2)	2.21 (2)	2.881 (2)	141 (2)
O3*W*—H3*W*1⋯O1*S*^i^	0.82 (2)	1.90 (2)	2.6982 (17)	163 (2)
O3*W*—H3*W*2⋯O4*W*^ii^	0.80 (2)	2.03 (2)	2.811 (2)	169 (2)
O4*W*—H4*W*1⋯I^vi^	0.82 (2)	2.67 (2)	3.4897 (18)	176 (3)
O4*W*—H4*W*2⋯O2*W*	0.82 (2)	1.93 (2)	2.728 (2)	164 (3)

Symmetry codes: (i) 
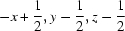
; (ii) 
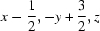
; (iii) 
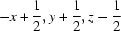
; (iv) 
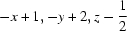
; (v) 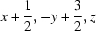; (vi) 
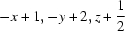
.

**Fig. 1.**
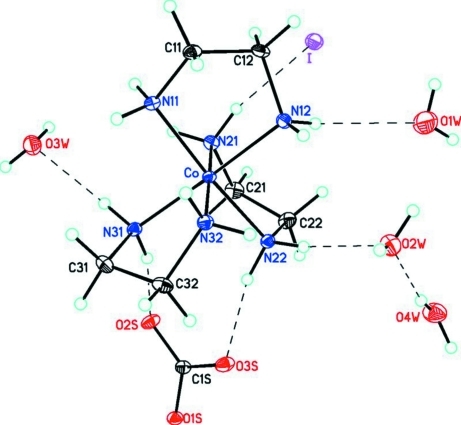
Molecular structure of C7H32CoIN6O7, showing the atom labeling scheme and 50% probability displacement ellipsoids. Dashed lines indicate O–H···O, N–H···H, N–H···I and O–H···I intermolecular hydrogen bonds (Table 1) in the asymmetric unit.

**Fig. 2.**
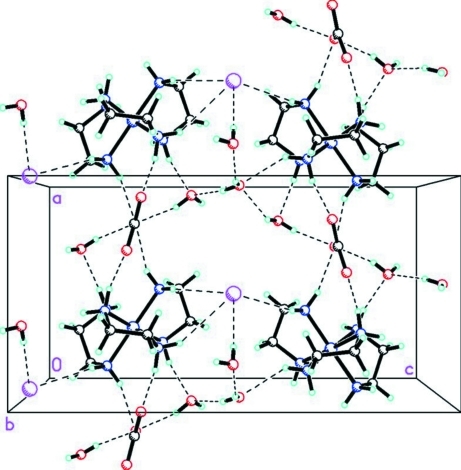
Packing diagram of the C7H32CoIN6O7 viewed down the b axis. Dashed lines indicate O–H···O, N–H···H, N–H···I and O–H···I intermolecular hydrogen bond interactions (Table 1).

## supplementary crystallographic information

### Comment

[Ni(en)_3_]^2+^ and [Zn(en)_3_]^2+^ react with MX (*M* = K or NH4, *X* = SCN– or SeCN–) to form double salts, [Ni(en)_3_](SCN)_2_.NH_4_(SCN) (Dvorkin *et al.*, 1991) and [Ni(en)_3_](SeCN)_2_.K(SeCN) (Farago *et al.*, 1967) or [Zn(en)_3_](SCN)_2_.K(SCN) (Dvorkin *et al.*, 1989). Structural studies of these thiocyanate double salts reveal a linear polymeric anion, [(*M*(SCN)_3_)^2-^]_n_. The reaction of [Co(en)_3_]^3+^ with potassium cyanate (Brewer & Butcher, 2009) was conducted to determine if the [(K(OCN)_3_)^2-^]_n_ ion could be formed and isolated as its salt with the [Co(en)_3_]^3+^ cation. Analysis of the reaction product by single-crystal diffraction revealed the [Co(en)_3_]^3+^ cation and carbonate and iodide ions. This suggests hydroysis of cyanate as it is the only source of a carbon atom in the reaction mixture other than the ethylenediamine ligand, (*i.e.*, [Co(en)_3_](I)_3_ + KOCN + 2H_2_O → [Co(en)_3_](CO_3_)(I).4H_2_O + NH_4_I + KI). The hydrolysis of cyanate to give carbonate has been observed with nickel (Seifer & Tarasova, 1982) and yttrium (Seifer *et al.*, 1981) at elevated temperatures. In addition HNCO (Piazzesi *et al.*, 2007) was hydrolyzed at elevated temperatures in the presence of solid catalysts. However, the present reaction takes place at room temperature. The thermodynamics of the outer sphere solution interaction of [Co(en)_3_]^3+^ with the carbonate ion (added as a carbonate salt) have been reported (Mironov, *et al.*, 1973, 1976). Similar structures containing the [Co(en)_3_]^3+^ cation have been reported (Brouty *et al.* 1976; Liu *et al.*, 1995). Additional related structures have been also been reported (Lappin, *et al.*, 1993; Mizuta *et al.*, 1988). Hence in continuation with our studies of the potential catalytic role of [Co(en)_3_]^3+^ in the hydrolysis of amides and urea and the relationship to urease this new tris(ethane-1,2-diamine-K^2^ N,*N*')cobalt(III) carbonate iodide tetrahydrate compound is synthesized and its crystal structure is reported.

The title compound crystallizes with a [Co(en)_3_]^3+^ cation, (CO_3_)^2-^ and I^-^ anions and four water molecules in the asymmetric unit (Fig. 1). In the cation the three rings formed by the ethylenediamine units and Co^3+^metal ion are in slightly distorted twist conformations with C11—C12, C21—C22 and C31—C32 being twisted within rings 1 (Co/N11/C11/C12/N12), 2 (Co/N21/C21/C22/N22) and 3 (Co/N31/C31/C32/N32), respectively. Numerous O–H···O, N–H···H, N–H···I and O–H···I intermolecular hydrogen bonds (Table 1) between the cation and two anions in concert with the four water molecules dominate the crystal packing and create a supramolecular infinite three-dimensional framework that extends throughout the crystalline lattice (Fig. 2).

### Experimental

[Co(en)_3_]I_3_ was prepared as described previously (Broomhead *et al.*, 1960). [Co(en)_3_]I_3_ was reacted with an excess of KOCN in water. The red blockish crystals were removed a week later by filtration.

### Refinement

The H atoms were placed in geometrically idealized positions and constrained to ride on their parent atoms with N—H = 0.92 Å, and C—H = 0.99 Å and with *U*_iso_(H) = 1.17–1.20 *U*_eq_(N) and *U*_iso_(H) = 1.19–1.21 *U*_eq_(C). H atoms on the water molecules were located by Fourier maps,   and refined isotropically with O-H restrained to 0.82 (2)Å and H..H restrained to 1.297 (2)Å and *U*_iso_(H) =  1.50 *U*_eq_(O).

### Crystal data

**Table:**

[Co(C_2_H_8_N_2_)_3_](CO_3_)I·4H_2_O	*F*(000) = 1008
*M**_r_* = 498.22	*D*_x_ = 1.812 Mg m^−^^3^
Orthorhombic, *P**n**a*2_1_	Mo *K*α radiation, λ = 0.71073 Å
Hall symbol: P 2c -2n	Cell parameters from 14059 reflections
*a* = 16.6907 (2) Å	θ = 4.6–34.7°
*b* = 8.7031 (1) Å	µ = 2.67 mm^−^^1^
*c* = 12.5718 (2) Å	*T* = 123 K
*V* = 1826.19 (4) Å^3^	Chunk, orange
*Z* = 4	0.52 × 0.46 × 0.35 mm

### Data collection

**Table:**

Oxford Diffraction Gemini R diffractometer	7440 independent reflections
Radiation source: Enhance (Mo) X-ray Source	6109 reflections with *I* > 2σ(*I*)
graphite	*R*_int_ = 0.028
Detector resolution: 10.5081 pixels mm^-1^	θ_max_ = 34.9°, θ_min_ = 4.6°
φ and ω scans	*h* = −26→26
Absorption correction: multi-scan (*CrysAlis RED*; Oxford Diffraction, 2007)	*k* = −13→13
*T*_min_ = 0.737, *T*_max_ = 1.000	*l* = −20→19
25329 measured reflections	

### Refinement

**Table:**

Refinement on *F*^2^	Hydrogen site location: inferred from neighbouring sites
Least-squares matrix: full	H atoms treated by a mixture of independent and constrained refinement
*R*[*F*^2^ > 2σ(*F*^2^)] = 0.023	*w* = 1/[σ^2^(*F*_o_^2^) + (0.0247*P*)^2^] where *P* = (*F*_o_^2^ + 2*F*_c_^2^)/3
*wR*(*F*^2^) = 0.048	(Δ/σ)_max_ = 0.004
*S* = 0.97	Δρ_max_ = 0.50 e Å^−^^3^
7440 reflections	Δρ_min_ = −0.52 e Å^−^^3^
224 parameters	Extinction correction: *SHELXL97* (Sheldrick, 2008), Fc^*^=kFc[1+0.001xFc^2^λ^3^/sin(2θ)]^-1/4^
13 restraints	Extinction coefficient: 0.0039 (3)
Primary atom site location: structure-invariant direct methods	Absolute structure: Flack (1983), 3466 Friedel pairs
Secondary atom site location: difference Fourier map	Flack parameter: 0.034 (8)

### Special details

**Table:**

Geometry. All e.s.d.'s (except the e.s.d. in the dihedral angle between two l.s. planes) are estimated using the full covariance matrix. The cell e.s.d.'s are taken into account individually in the estimation of e.s.d.'s in distances, angles and torsion angles; correlations between e.s.d.'s in cell parameters are only used when they are defined by crystal symmetry. An approximate (isotropic) treatment of cell e.s.d.'s is used for estimating e.s.d.'s involving l.s. planes.
Refinement. Refinement of *F*^2^ against ALL reflections. The weighted *R*-factor *wR* and goodness of fit *S* are based on *F*^2^, conventional *R*-factors *R* are based on *F*, with *F* set to zero for negative *F*^2^. The threshold expression of *F*^2^ > σ(*F*^2^) is used only for calculating *R*-factors(gt) *etc*. and is not relevant to the choice of reflections for refinement. *R*-factors based on *F*^2^ are statistically about twice as large as those based on *F*, and *R*- factors based on ALL data will be even larger.

### Fractional atomic coordinates and isotropic or equivalent isotropic displacement parameters (Å^2^)

**Table:**

	*x*	*y*	*z*	*U*_iso_*/*U*_eq_	
Co	0.253677 (10)	0.80188 (2)	0.618078 (18)	0.01164 (4)	
N11	0.18624 (8)	0.71681 (17)	0.50344 (11)	0.0151 (3)	
H11A	0.1933	0.6121	0.4995	0.018*	
H11B	0.1331	0.7358	0.5179	0.018*	
N12	0.31791 (8)	0.89092 (17)	0.50246 (11)	0.0158 (3)	
H12A	0.3066	0.9939	0.4957	0.019*	
H12B	0.3716	0.8805	0.5174	0.019*	
N21	0.31973 (7)	0.61537 (14)	0.62246 (13)	0.0166 (2)	
H21A	0.2875	0.5297	0.6213	0.020*	
H21B	0.3529	0.6121	0.5641	0.020*	
N22	0.32782 (8)	0.88267 (16)	0.72458 (11)	0.0153 (3)	
H22A	0.3444	0.9794	0.7050	0.018*	
H22B	0.3023	0.8900	0.7892	0.018*	
N31	0.18243 (8)	0.71917 (17)	0.72761 (12)	0.0153 (3)	
H31A	0.1657	0.6224	0.7083	0.018*	
H31B	0.2097	0.7114	0.7910	0.018*	
N32	0.18705 (7)	0.98751 (14)	0.62582 (13)	0.0161 (2)	
H32A	0.2190	1.0736	0.6239	0.019*	
H32B	0.1526	0.9909	0.5687	0.019*	
C11	0.20837 (10)	0.7882 (2)	0.40106 (13)	0.0185 (3)	
H11C	0.1811	0.8885	0.3928	0.022*	
H11D	0.1925	0.7210	0.3412	0.022*	
C12	0.29845 (10)	0.8098 (2)	0.40232 (13)	0.0189 (3)	
H12C	0.3259	0.7091	0.3998	0.023*	
H12D	0.3158	0.8714	0.3402	0.023*	
C21	0.36832 (10)	0.6175 (2)	0.72172 (14)	0.0226 (4)	
H21C	0.4138	0.5449	0.7160	0.027*	
H21D	0.3350	0.5877	0.7836	0.027*	
C22	0.39832 (9)	0.7795 (2)	0.73421 (14)	0.0222 (3)	
H22C	0.4241	0.7927	0.8045	0.027*	
H22D	0.4382	0.8035	0.6783	0.027*	
C31	0.11178 (10)	0.8214 (2)	0.74117 (14)	0.0218 (3)	
H31C	0.0884	0.8080	0.8130	0.026*	
H31D	0.0702	0.7965	0.6877	0.026*	
C32	0.14064 (10)	0.9841 (2)	0.72663 (17)	0.0218 (3)	
H32C	0.0946	1.0555	0.7225	0.026*	
H32D	0.1750	1.0151	0.7871	0.026*	
O1S	0.25421 (6)	0.80615 (15)	1.08978 (9)	0.0194 (2)	
O2S	0.23978 (8)	0.67827 (14)	0.93676 (10)	0.0232 (3)	
O3S	0.27443 (8)	0.92499 (15)	0.93456 (10)	0.0244 (3)	
C1S	0.25620 (8)	0.80301 (18)	0.98495 (13)	0.0159 (3)	
O1W	0.48655 (10)	1.0158 (3)	0.4929 (2)	0.0495 (6)	
H1W1	0.4953 (16)	1.003 (5)	0.4303 (16)	0.074*	
H1W2	0.5264 (15)	1.041 (4)	0.522 (2)	0.074*	
O2W	0.38714 (8)	1.18109 (17)	0.64128 (11)	0.0285 (3)	
H2W1	0.3484 (10)	1.228 (3)	0.625 (2)	0.043*	
H2W2	0.4135 (11)	1.177 (3)	0.5871 (16)	0.043*	
O3W	0.10686 (8)	0.44224 (16)	0.64602 (12)	0.0281 (3)	
H3W1	0.1450 (11)	0.385 (3)	0.635 (2)	0.042*	
H3W2	0.0811 (12)	0.406 (3)	0.6930 (17)	0.042*	
O4W	0.49798 (8)	1.1631 (2)	0.80022 (14)	0.0363 (4)	
H4W1	0.4993 (11)	1.238 (3)	0.839 (3)	0.054*	
H4W2	0.4597 (13)	1.180 (3)	0.7605 (19)	0.054*	
I	0.500266 (6)	0.526338 (14)	0.47581 (2)	0.02884 (4)	

### Atomic displacement parameters (Å^2^)

**Table:**

	*U*^11^	*U*^22^	*U*^33^	*U*^12^	*U*^13^	*U*^23^
Co	0.01398 (8)	0.01044 (7)	0.01049 (7)	0.00031 (6)	0.00016 (8)	−0.00020 (9)
N11	0.0174 (6)	0.0146 (7)	0.0132 (6)	0.0009 (5)	−0.0016 (5)	−0.0011 (5)
N12	0.0166 (6)	0.0155 (7)	0.0152 (6)	0.0000 (5)	0.0015 (4)	0.0001 (5)
N21	0.0190 (5)	0.0138 (6)	0.0170 (6)	0.0029 (4)	0.0011 (6)	0.0012 (6)
N22	0.0173 (6)	0.0136 (7)	0.0151 (6)	−0.0006 (5)	0.0001 (5)	−0.0009 (5)
N31	0.0202 (6)	0.0118 (7)	0.0139 (6)	−0.0018 (5)	0.0007 (5)	−0.0002 (5)
N32	0.0184 (5)	0.0151 (6)	0.0149 (6)	0.0000 (4)	0.0004 (6)	−0.0007 (5)
C11	0.0239 (8)	0.0189 (8)	0.0125 (7)	0.0003 (6)	−0.0022 (6)	0.0009 (6)
C12	0.0246 (8)	0.0210 (9)	0.0111 (7)	0.0010 (6)	0.0032 (6)	−0.0012 (6)
C21	0.0249 (8)	0.0232 (10)	0.0199 (8)	0.0072 (7)	−0.0038 (7)	0.0039 (6)
C22	0.0178 (7)	0.0265 (9)	0.0223 (8)	0.0036 (7)	−0.0060 (6)	−0.0027 (7)
C31	0.0193 (7)	0.0263 (9)	0.0197 (8)	0.0011 (6)	0.0058 (6)	0.0005 (7)
C32	0.0232 (7)	0.0226 (9)	0.0195 (7)	0.0065 (7)	0.0042 (8)	−0.0033 (6)
O1S	0.0242 (5)	0.0213 (6)	0.0129 (5)	−0.0016 (4)	−0.0006 (4)	−0.0013 (4)
O2S	0.0389 (7)	0.0136 (6)	0.0172 (6)	−0.0040 (5)	−0.0030 (5)	−0.0013 (4)
O3S	0.0428 (7)	0.0138 (6)	0.0166 (5)	−0.0044 (5)	0.0023 (5)	0.0001 (5)
C1S	0.0195 (6)	0.0146 (6)	0.0136 (6)	0.0023 (5)	0.0001 (7)	0.0005 (7)
O1W	0.0317 (8)	0.0663 (12)	0.0506 (18)	−0.0069 (7)	0.0049 (8)	−0.0220 (10)
O2W	0.0264 (6)	0.0335 (8)	0.0257 (7)	0.0008 (5)	−0.0022 (5)	0.0036 (6)
O3W	0.0262 (6)	0.0259 (7)	0.0321 (8)	−0.0011 (5)	0.0036 (5)	−0.0006 (6)
O4W	0.0326 (8)	0.0494 (10)	0.0269 (7)	0.0031 (6)	−0.0040 (6)	−0.0047 (7)
I	0.02008 (5)	0.04341 (7)	0.02305 (5)	−0.00295 (5)	−0.00117 (4)	−0.00428 (9)

### Geometric parameters (Å, °)

**Table:**

Co—N22	1.9541 (14)	C11—H11C	0.9900
Co—N31	1.9566 (14)	C11—H11D	0.9900
Co—N21	1.9630 (12)	C12—H12C	0.9900
Co—N32	1.9637 (12)	C12—H12D	0.9900
Co—N12	1.9654 (14)	C21—C22	1.505 (3)
Co—N11	1.9729 (14)	C21—H21C	0.9900
N11—C11	1.476 (2)	C21—H21D	0.9900
N11—H11A	0.9200	C22—H22C	0.9900
N11—H11B	0.9201	C22—H22D	0.9900
N12—C12	1.479 (2)	C31—C32	1.507 (3)
N12—H12A	0.9200	C31—H31C	0.9900
N12—H12B	0.9200	C31—H31D	0.9900
N21—C21	1.488 (2)	C32—H32C	0.9900
N21—H21A	0.9201	C32—H32D	0.9900
N21—H21B	0.9200	O1S—C1S	1.3186 (19)
N22—C22	1.485 (2)	O2S—C1S	1.273 (2)
N22—H22A	0.9201	O3S—C1S	1.273 (2)
N22—H22B	0.9200	O1W—H1W1	0.808 (18)
N31—C31	1.487 (2)	O1W—H1W2	0.788 (17)
N31—H31A	0.9199	O2W—H2W1	0.791 (15)
N31—H31B	0.9201	O2W—H2W2	0.811 (15)
N32—C32	1.486 (2)	O3W—H3W1	0.820 (15)
N32—H32A	0.9200	O3W—H3W2	0.795 (15)
N32—H32B	0.9201	O4W—H4W1	0.818 (17)
C11—C12	1.515 (2)	O4W—H4W2	0.824 (16)
			
N22—Co—N31	92.01 (6)	Co—N32—H32A	109.9
N22—Co—N21	85.56 (6)	C32—N32—H32B	109.9
N31—Co—N21	90.99 (6)	Co—N32—H32B	109.9
N22—Co—N32	91.64 (6)	H32A—N32—H32B	108.3
N31—Co—N32	85.62 (6)	N11—C11—C12	106.94 (12)
N21—Co—N32	175.53 (8)	N11—C11—H11C	110.3
N22—Co—N12	91.11 (5)	C12—C11—H11C	110.3
N31—Co—N12	175.62 (6)	N11—C11—H11D	110.3
N21—Co—N12	92.32 (6)	C12—C11—H11D	110.3
N32—Co—N12	91.21 (6)	H11C—C11—H11D	108.6
N22—Co—N11	175.48 (6)	N12—C12—C11	106.62 (13)
N31—Co—N11	91.68 (5)	N12—C12—H12C	110.4
N21—Co—N11	91.75 (6)	C11—C12—H12C	110.4
N32—Co—N11	91.25 (6)	N12—C12—H12D	110.4
N12—Co—N11	85.35 (6)	C11—C12—H12D	110.4
C11—N11—Co	109.64 (10)	H12C—C12—H12D	108.6
C11—N11—H11A	109.7	N21—C21—C22	106.28 (14)
Co—N11—H11A	109.7	N21—C21—H21C	110.5
C11—N11—H11B	109.7	C22—C21—H21C	110.5
Co—N11—H11B	109.7	N21—C21—H21D	110.5
H11A—N11—H11B	108.2	C22—C21—H21D	110.5
C12—N12—Co	108.75 (10)	H21C—C21—H21D	108.7
C12—N12—H12A	109.9	N22—C22—C21	107.12 (13)
Co—N12—H12A	109.9	N22—C22—H22C	110.3
C12—N12—H12B	109.9	C21—C22—H22C	110.3
Co—N12—H12B	109.9	N22—C22—H22D	110.3
H12A—N12—H12B	108.3	C21—C22—H22D	110.3
C21—N21—Co	108.63 (11)	H22C—C22—H22D	108.5
C21—N21—H21A	110.0	N31—C31—C32	107.14 (13)
Co—N21—H21A	110.0	N31—C31—H31C	110.3
C21—N21—H21B	110.0	C32—C31—H31C	110.3
Co—N21—H21B	110.0	N31—C31—H31D	110.3
H21A—N21—H21B	108.3	C32—C31—H31D	110.3
C22—N22—Co	109.88 (10)	H31C—C31—H31D	108.5
C22—N22—H22A	109.7	N32—C32—C31	106.79 (15)
Co—N22—H22A	109.7	N32—C32—H32C	110.4
C22—N22—H22B	109.7	C31—C32—H32C	110.4
Co—N22—H22B	109.7	N32—C32—H32D	110.4
H22A—N22—H22B	108.2	C31—C32—H32D	110.4
C31—N31—Co	110.04 (11)	H32C—C32—H32D	108.6
C31—N31—H31A	109.7	O2S—C1S—O3S	121.71 (16)
Co—N31—H31A	109.7	O2S—C1S—O1S	119.20 (15)
C31—N31—H31B	109.7	O3S—C1S—O1S	119.08 (15)
Co—N31—H31B	109.7	H1W1—O1W—H1W2	109 (2)
H31A—N31—H31B	108.2	H2W1—O2W—H2W2	104 (2)
C32—N32—Co	108.74 (11)	H3W1—O3W—H3W2	108 (2)
C32—N32—H32A	109.9	H4W1—O4W—H4W2	104 (2)
			
N31—Co—N11—C11	165.33 (12)	N21—Co—N31—C31	−172.33 (12)
N21—Co—N11—C11	−103.63 (11)	N32—Co—N31—C31	10.59 (11)
N32—Co—N11—C11	79.67 (11)	N11—Co—N31—C31	−80.54 (12)
N12—Co—N11—C11	−11.44 (11)	N22—Co—N32—C32	−74.29 (11)
N22—Co—N12—C12	159.63 (11)	N31—Co—N32—C32	17.60 (11)
N21—Co—N12—C12	74.03 (11)	N12—Co—N32—C32	−165.44 (11)
N32—Co—N12—C12	−108.70 (11)	N11—Co—N32—C32	109.19 (11)
N11—Co—N12—C12	−17.54 (11)	Co—N11—C11—C12	37.13 (14)
N22—Co—N21—C21	17.86 (11)	Co—N12—C12—C11	41.99 (14)
N31—Co—N21—C21	−74.07 (11)	N11—C11—C12—N12	−51.59 (16)
N12—Co—N21—C21	108.80 (11)	Co—N21—C21—C22	−42.12 (15)
N11—Co—N21—C21	−165.78 (11)	Co—N22—C22—C21	−36.80 (16)
N31—Co—N22—C22	101.65 (11)	N21—C21—C22—N22	51.17 (17)
N21—Co—N22—C22	10.81 (11)	Co—N31—C31—C32	−36.00 (17)
N32—Co—N22—C22	−172.68 (11)	Co—N32—C32—C31	−41.51 (15)
N12—Co—N22—C22	−81.43 (11)	N31—C31—C32—N32	50.28 (18)
N22—Co—N31—C31	102.08 (11)		

### Hydrogen-bond geometry (Å, °)

**Table:**

*D*—H···*A*	*D*—H	H···*A*	*D*···*A*	*D*—H···*A*
N11—H11A···O3S^i^	0.92	1.90	2.7625 (19)	155.
N11—H11B···I^ii^	0.92	3.08	3.8409 (14)	141.
N12—H12A···O2S^iii^	0.92	1.93	2.8042 (19)	158.
N12—H12B···O1W	0.92	2.27	3.020 (2)	138.
N21—H21A···O1S^i^	0.92	2.10	2.9891 (18)	161.
N21—H21B···I	0.92	2.80	3.6165 (13)	149.
N22—H22A···O2W	0.92	2.06	2.970 (2)	172.
N22—H22B···O3S	0.92	1.91	2.8104 (19)	166.
N31—H31A···O3W	0.92	2.01	2.907 (2)	165.
N31—H31B···O2S	0.92	1.92	2.821 (2)	165.
N32—H32A···O1S^iii^	0.92	2.12	2.9760 (18)	155.
N32—H32A···O2S^iii^	0.92	2.61	3.146 (2)	117.
N32—H32B···I^ii^	0.92	2.80	3.6456 (13)	153.
O1W—H1W1···O4W^iv^	0.81 (2)	2.19 (3)	2.892 (3)	146 (4)
O1W—H1W2···O3W^v^	0.79 (2)	2.07 (2)	2.805 (3)	156 (3)
O2W—H2W1···O1S^iii^	0.79 (2)	1.90 (2)	2.6775 (17)	170 (2)
O2W—H2W2···O1W	0.81 (2)	2.21 (2)	2.881 (2)	141 (2)
O3W—H3W1···O1S^i^	0.82 (2)	1.90 (2)	2.6982 (17)	163 (2)
O3W—H3W2···O4W^ii^	0.80 (2)	2.03 (2)	2.811 (2)	169 (2)
O4W—H4W1···I^vi^	0.82 (2)	2.67 (2)	3.4897 (18)	176 (3)
O4W—H4W2···O2W	0.82 (2)	1.93 (2)	2.728 (2)	164 (3)

Symmetry codes: (i) −*x*+1/2, *y*−1/2, *z*−1/2; (ii) *x*−1/2, −*y*+3/2, *z*; (iii) −*x*+1/2, *y*+1/2, *z*−1/2; (iv) −*x*+1, −*y*+2, *z*−1/2; (v) *x*+1/2, −*y*+3/2, *z*; (vi) −*x*+1, −*y*+2, *z*+1/2.
